# Chemical and Bioactive Characterization of Spanish and Belgian Apple Pomace for Its Potential Use as a Novel Dermocosmetic Formulation

**DOI:** 10.3390/foods10081949

**Published:** 2021-08-21

**Authors:** Ana Alvarez Arraibi, Ângela Liberal, Maria Inês Dias, Maria José Alves, Isabel C. F. R. Ferreira, Lillian Barros, João C. M. Barreira

**Affiliations:** Centro de Investigação de Montanha (CIMO), Instituto Politécnico de Bragança, Campus de Santa Apolónia, 5300-253 Bragança, Portugal; ana_arraibi@hotmail.com (A.A.A.); angela_lib@hotmail.com (Â.L.); maria.ines@ipb.pt (M.I.D.); martia.alves@ipb.pt (M.J.A.); iferreira@ipb.pt (I.C.F.R.F.); lillian@ipb.pt (L.B.)

**Keywords:** apple pomace, phenolic compounds, cosmeceuticals, anti-acne activity, natural hydrogel formulations

## Abstract

Currently, there is a general trend towards reutilizing industrial by-products that would otherwise be discarded or considered as waste, aiming to explore them as alternative sources of valuable compounds. The apple pomace remaining from cider and apple juice industries represents a high-potential source of bioactive compounds with putative application in food or pharmaceutical-related products. Accordingly, the work reported herein was conducted to characterize the phenolic compounds in apple pomace from Belgium and Spain, as well as to evaluate its chemical composition and particular types of bioactivity. As a proof of concept, a new hydrogel was prepared, incorporated with the bioactive compounds and pectin extracted from apple pomace, aiming to obtain the most organic formulation possible. Independently of the extracting agent, it became evident that using lyophilization as the drying step is a better choice than thermal processes as it yielded a richer phenolic profile (fifteen individual compounds), with 5-*O*-caffeoylquinic acid as the major compound (66 to 114 mg/100 g dw) in Belgian samples. In general, the hydroethanolic extracts showed the strongest antioxidant and antimicrobial (particularly against *Propionibacterium acnes*: MIC = 2.5 mg/mL) activities. This result, together with the lipid nature of human skin, led it to be chosen as the extract type to be incorporated in the hydrogel. In general, apple pomace stood out as a valuable source of bioactive compounds, especially polyphenols and pectin, with good potential to be incorporated in dermal formulations.

## 1. Introduction

The growing consumer consciousness about health complications associated with the use of conventional cosmetics, together with the need for a better understanding of the skin’s physiology, has sponsored the demand for innovative cosmetic products based on natural substances [1]. In fact, since ancient times, the plant kingdom has been the basis of natural constituents with potential effects on protection, healing, and upkeep of the beauty of human skin through the use of herbal and seed extracts or aromatized waters [2]. Nowadays, the upward association between beauty, health, and well-being has boosted the search for so-called “natural cosmetics”, the market for which, although still relatively small, is increasing much faster than the cosmetics industry in general [2,3,4].

Usually, conventional cosmetics cover a wide range of chemical substances, most of which are not protected by the regulatory scope of the Food and Drug Administration (FDA) or the European Medicines Agency (EMA), and only a few (e.g., parabens, phthalates, polycyclic aromatic hydrocarbons, siloxanes, and triclosan) have already been assessed for their potential harmful effects on human health [5]. In turn, herbal-based products hold several potentially valuable active compounds that can be useful in the handling of different skin illnesses [2,6]. Obtaining these natural substances can be favorable when natural matrices are widely available, as in the case of industrially processed fruit by-products, which represents an ecological approach to reusing the large amounts of waste generated in this type of industry and at reduced prices [7].

Apple (*Malus* sp., *Rosaceae*) is one of the most popular and highly consumed fruits worldwide, being known for its valuable effects in preventing cardiovascular and respiratory diseases, diabetes, obesity, and cancer [7]. Most harvested apples are processed and converted into juice and cider, an industrial activity that produces a large sum of underutilized by-products. The apple pomace that remains after juice extraction and cider production (circa ¼ of the fresh fruit weight) consists of leftover flesh, peels, seeds, and stems of different apple varieties [8,9]. Despite being naturally considered as waste, these residual materials are rich in bioactive compounds, particularly polyphenols, fibers, vitamins, and carotenoids [10]. Although some polyphenols are transferred to juice during the processing step, most remain in apple peel and, consequently, in apple pomace. Phytochemicals and active compounds present in the latter also include dietary fiber, pectin, triterpenoids, and volatile compounds. Pectin is known for its application as a gelling agent, being employed in different industrial sectors (food, pharmaceutics, and cosmetics), playing also several biological and physiological functions in human organisms. These functional properties make pectin a promising nutraceutical with low toxicity and production cost [11,12,13]. 

Even though apple pomace by-products present a high potential in several sectors, these are still far from being used at full potential, especially in the food and pharmaceutical industries [9,14]. Its bioactive compounds can be exploited as cosmeceuticals which, in addition to their inherent application, can be used to promote skin health. The search for these types of cosmetic products presents itself as a challenge in the current cosmetic industry. Among the polyphenols identified in apple pomace, phloretin and phlorizin seem to have the greatest potential as natural alternatives to synthetic antimicrobials and antioxidants [15]. In addition to its anti-aging effects, identified by Park et al. [16], an in vitro study also demonstrated the inhibitory capacity of a polyphenolic apple extract on the fat type produced in sebaceous cells, suggesting that these secondary metabolites might regulate sebum production, relieve skin diseases such as acne [17], and reduce dermal inflammation, a capacity attributed to their inherent antioxidant assets [18].

Thus, the present study intends to explore and describe the phenolic composition and bioactivity (antioxidant and antimicrobial) of apple pomace to validate its potential as an innovative source of cosmeceuticals to be included in new natural formulations, adding value to this material currently considered as industrial bio-waste. In addition to this main purpose, an additional preliminary characterization of its proximate composition was also performed to assess potential food applications. Moreover, instead of using typical commercial gelling agents (such as carbopol 940), we intended to isolate pectin from apple pomace by hot acid extraction, thus using this by-product considering two distinct aims. 

## 2. Material and Methods

### 2.1. Standards and Reagents

HPLC-grade acetonitrile was purchased from Fisher Scientific (Lisbon, Portugal). Standards of gallic acid and catechin were acquired from Sigma (St. Louis, MO, USA), 2,2-diphenyl-1-picrylhydrazyl (DPPH) and β-carotene from Alfa Aesar (Ward Hill, MA, USA), and phenolic compounds from Extrasynthese (Genay, France). Muller–Hinton broth (MHB), tryptic soy broth (TSB), blood agar with 7% sheep blood, and MacConkey agar plates were obtained from Biomerieux (Marcyl’Etoile, France). The dye *p*-iodonitrotetrazolium chloride (used as a microbial growth indicator) was provided by Sigma-Aldrich (St. Louis, MO, USA), while propanediol, citric acid, and triethanolamine were from Sigma (St. Louis, MO, USA). Ethanol and all other solvents and chemicals were of analytical grade and provided by Merck (Darmstadt, Germany). A Milli-Q water purification system (TGI Pure Water Systems, Greenville, SC, USA) was used to treat water.

### 2.2. Samples

Apple pomace samples were kindly supplied by Corporación Hijos de la Rivera S.L. (La Coruña, Spain) and Tuinsappen Lombarts Calville (Ypres, Belgium). The pomaces were obtained from a mixture of apple varieties harvested during the 2017 season. Samples from Spain consisted mainly of a mixture of Rabosa, Pero, Príncipe, Verdeña, Perezosa, and De la Riega varieties. The Spanish pomace samples were immediately frozen after the pressing step in the beginning of November and kept at −80 °C until lyophilization. Samples obtained from the Belgian company consisted of approximately 25 different regional “old varieties”, such as Jacques Lebel, Keuleman, Lombarts Calville, Reinette de Flandre, Quastresdouble, Collapuis, and President van Dievoet. In turn, the Belgian pomace samples were dried at 50 °C immediately after the pressing step in December 2017, and two different pomace types were provided: one from cider production and the other from juice production, where the varieties were sweeter. 

Both samples were then ground into a fine powder and stored protected from light and humidity until further analysis.

### 2.3. Extract Preparation 

Decoction, hydromethanolic (methanol:water, 80:20, *v/v*), hydroethanolic (ethanol: water, 80:20, *v/v*), and ethanolic extracts from both apple pomace samples were prepared, and the extracts were used for determination of phenolic compounds and biological activities. Briefly, for decoction extracts, each sample (~1 g) was kept (5 min) in ebullition in 100 mL of distilled water and filtered through Whatman filter paper No 4. The obtained decoctions were freeze-dried (FreeZone 4.5, Labconco, Kansas City, MO, USA) [19]. The remaining extracts were prepared by macerating the dry material with each corresponding solvent [20]. Briefly, ~1 g of each sample was extracted (1 h, 25 ℃, 150 rpm) twice with 30 mL of each solvent and filtered through Whatman No. 4 paper. The alcohol solvents were removed using a rotary evaporator (Büchi R-210, Flawil, Switzerland); the remaining water was lyophilized (FreeZone 4.5 model 7750031, Labconco, Kansas City, MO, USA).

### 2.4. Chemical Composition

#### 2.4.1. Proximate Composition and Energetic Value

Proximate composition: crude protein (macro-Kjeldahl method, N × 6.25, model Pro-Nitro-A, JP Selecta, Barcelona), crude fat (Soxhlet extraction, petroleum ether, 7 h), and ash (incineration, 550 ± 5 ℃) were determined and expressed in g/100 g dry sample [21]. Total carbohydrates content was calculated by difference:

Total carbohydrates (g/100 g) = 100 − (g_fat_ + g_ash_ + g_proteins_) (1)

The corresponding energy was calculated according to the Atwater system:

Energy (kcal/100 g) = 4 × (g proteins + g carbohydrates) + 9 × (g fat)(2)

#### 2.4.2. Sugars

The method employed by Barros et al. [20] was followed. Sugars were identified using high-performance liquid chromatography with a refraction index detector (HPLC-RI; Knauer, Smartline 1000 and Smartline 2300 systems, respectively) under the previously optimized chromatographic conditions. The identification step was performed by comparing the relative retention time (Rt) of the peaks with authentic standards. Quantification (g/100 g dw) was based on the internal standard method (IS: melezitose, Sigma-Aldrich, St. Louis, MO, USA) using the Clarity Software (Data Apex, Prague, Czech Republic).

#### 2.4.3. Phenolic Compounds 

The phenolic profile was evaluated (Dionex Ultimate 3000 UPLC, Thermo Scientific, San Jose, CA, USA) after redissolving each lyophilized extract in ethanol/water (80:20; *v/v*) to a final concentration of 10 mg/mL (for decoctions and hydromethanolic extracts) or 20 mg/mL (for hydroethanolic and ethanolic extracts). A DAD detector (280 and 370 nm as the preferred wavelengths) coupled to an electrospray ionization mass detector (LC-DAD-ESI/MSn) was used. The selected column was a Waters Spherisorb S3 ODS-2 C18 (3 µm, 4.6 × 150 mm, Waters, Milford, MA, USA) functioning at 35 ℃. Compounds were eluted with a gradient mixture of 0.1% formic acid in water and acetonitrile. MS analysis was performed in negative mode (Linear Ion Trap LTQ XL mass spectrometer, ThermoFinnigan, San Jose, CA, USA) using an electrospray ionization source (ESI). Phenolic compounds were identified based on their chromatographic behavior, spectra, and UV–Vis mass, either by comparison with authentic standards or available data from similar studies using the Xcalibur^®^ software (ThermoFinnigan, San Jose, CA, USA). Each compound concentration (mg/g of lyophilized extract) was quantified through calibration curves drawn from the UV signal of the corresponding (or most similar) standard compound [22].

### 2.5. Bioactive Properties

#### 2.5.1. Antioxidant Activity

The antioxidant activity of apple pomace extracts was assessed by the mean of three assays: (i) DPPH radical scavenging activity (ELX800 microplate reader, Bio-Tek Instruments, Inc.; Winooski, VT, USA) was calculated as the percentage of DPPH discoloration by comparing the absorbance at 515 nm:

[(A_DPPH_ − AS)/A_DPPH_] × 100 (3)

(ii) reducing power was evaluated as the capacity to convert Fe^3+^ into Fe^2+^ by measuring the absorbance at 690 nm; (iii) inhibition of β-carotene bleaching was evaluated through the β-carotene/linoleate assay, in which the neutralization of linoleate free radicals avoids β-carotene bleaching, indicated through the percentages obtained with the following formula:

(β-carotene absorbance after 2 h of assay/initial absorbance) × 100(4)

The results were expressed in EC_50_ values (sample concentration providing 50% of antioxidant activity or 0.5 of absorbance in the reducing power assay) [23].

#### 2.5.2. Antibacterial Activity

The microdilution method together with the rapid colorimetric *p*-iodonitrotetrazolium chloride (INT) assay was used. Stock solutions from each hydroalcoholic and ethanolic extract were prepared by dissolving 100 mg in 5 mL of TSB medium with 5% DMSO. The assay was performed with ATCC cultures (11827, Liofilchem, Italy)—*Propionibacterium acnes* (Gram-negative)—or with clinical isolates from patients hospitalized in the Local Health Unit of Bragança or the Central Hospital of Trás-os-Montes and Alto-Douro (Vila Real)—*Staphylococcus aureus* (Gram-positive) isolated from wound exudate, *Proteus mirabilis* (Gram-negative) from urine, and *Pseudomonas aeruginosa* (Gram-negative) from expectoration. The selected bacterial strains were used to screen the antimicrobial activity of the extracts in the study since they are typically found in human skin, with a special focus on *P. acnes*, linked to the skin disorder acne vulgaris. The activity indicator was presented as the minimal inhibition concentration (MIC). Ampicillin, methicillin, and streptomycin (for *P. acnes*) were used as positive controls.

### 2.6. Pectin Extraction

Pectin was obtained by hot acid extraction, following a previously established procedure [24], with minor modifications. In brief, ~50 g of frozen apple pomace was mixed with 200 mL of distilled water and heated to 90 °C under continuous stirring (350 rpm). Hydrochloric acid was added to decrease the pH to 1.5 or 3. The slurry was cooled using an ice bath for 30 min and the liquid portion was separated by centrifugation at 6000 rpm for 30 min at 15 °C. The supernatants were then vacuum filtered over a Buchner funnel with Whatman No. 4 filter paper. The remaining solids were centrifuged and filtered under the same conditions and the supernatants were combined, with a final pH readjustment to 3.5 with 1 M sodium hydroxide. The extract was then mixed with an equal volume of ethanol and stirred for 10 min at room temperature. The precipitate was separated by centrifugation under the same conditions mentioned above, dispersed in 500 mL of 70% ethanol, and stirred (10 min, 250 rpm, room temperature). The extracted pectin was freeze-dried (Labconco FreeZone 4.5, Kansas City, MO, USA) at −50 °C and 0.22 mbar for approximately 60 h. The yield was gravimetrically determined according to the following equation:

% 𝑝𝑒𝑐𝑡𝑖𝑛 = 𝑚_𝑝𝑒𝑐𝑡𝑖𝑛_/𝑚_𝑎𝑝𝑝_ × 100 (5)

where *m_pectin_* is the mass of pectin obtained and *m_app_* is the total mass of frozen apple pomace used in the extraction.

### 2.7. Dermocosmetic Gel Formulation

For the dermocosmetic gel formulation, pectin was redissolved in water by the direct addition method [25] and citric acid (300 mg) was added to create a more acidic environment and facilitate pectin dissolution. The solution was heated (60–100 ℃) under vigorous agitation for 20 min until complete dissolution. Then, 175 mg of the phenolic extract was dissolved in 500 μL of distilled water and 1 g of propylene glycol was used as a humectant agent. The resulting extract was added to the pectin gel and stirred at room temperature to obtain a homogeneous distribution of the phenolic extract. The pH was adjusted by adding 500 mg of triethanolamine, obtaining a final pH of 5.69. Other gels were prepared using 350 mg of phenolic extract and, in this case, citric acid was not added. A blank gel formulation (without phenolic extract) was also prepared for posterior comparison of bioactivities.

### 2.8. Bioactivity of the Dermocosmetic Gel 

Antioxidant and antimicrobial assays were performed for the different hydrogel formulations as previously described for the extracts (Section 2.4). In the DPPH antioxidant assay, the reaction was performed in a vial and not directly in the wells as usual since pectin precipitates at the bottom of the well, clogging the passage of light and consequently obstructing determination of the absorbance. 

### 2.9. Statistical Analysis

The analytical tests were performed in triplicate, and the corresponding results were expressed as mean values ± SD. Together with a homoscedasticity verification, the variance was compared using Tukey’s test (homoscedastic distributions) or Tamhane’s T2 test (heteroscedastic distributions). In cases with only two different available results (e.g., quercetin-*O*-hexoside II in Belgian apple pomace), Student’s *t*-test was applied instead using the same α = 0.05. The Shapiro–Wilk test and the Levene test were previously performed to verify the normal distribution of results and the homogeneity of variances. IBM SPSS Statistics for Windows, version 22.0, was used (IBM Corp., Armonk, NY, USA).

## 3. Results and Discussion

### 3.1. Proximate Composition

The results achieved for macronutrients in apple pomace samples are presented in Table 1. Since the samples provided by Tuinsappen Lombarts Calville (Belgium) were already dried by the company itself, the moisture content was not assessed. With respect to the samples provided by Corporación Hijos de la Rivera S.L. (Spain), they still retained a considerable water content (75.3 g/100 g fw), similar to that reported in a previous analytical study (71.5 to 78.6 g/100 g fw) performed by Gullón et al. [7], who investigated the average compositional values of nine apple pomace samples from different varieties. Carbohydrates were the major macronutrient, with values ranging from 93.0 to 94.2 g/100 g dw for the Spanish and Belgian (cider) samples, respectively. Moreover, according to the results attained for soluble sugars, carbohydrates were mainly present as individual sugars, particularly fructose and glucose and a small percentage of sucrose. The apple pomace from Belgian apple cultivars used in juice extraction presented the highest fructose content (55 g/100 g dw), which, in turn, was detected in its lowest quantity in the same Belgian cultivars used for cider production (42 g/100 g dw). Glucose and sucrose were both found in the highest amounts in the apple pomace from Spanish apple cultivars (26 and 7.4 g/100 g dw, respectively), thus presenting the highest sugar content among the studied samples. In both cases, the sugar content was similar to that detected in previous studies [7]. Since carbohydrates quantities detected among the samples under study were very alike, and those of sugars were not, it can be hypothesized that apple pomace from cider production with Belgian cultivars may have a higher percentage of total of fiber. Overall, the studied samples presented statistically significant differences (*p* < 0.05) regarding proteins, fat, and ash content, although the proximate profiles were very similar to those characterized in industrial apple pomace from a different company [26]. In general, apple pomace revealed an interesting nutritional potential, promoting its use in food products and offering a value-added alternative for other industries.

### 3.2. Phenolic Compounds

In order to investigate the effect of the extraction step on phenolic compounds’ yield and bioactivity performance, four different extracts of each apple pomace sample were prepared by maceration, using the same solid:liquid ratio (1:30) with different solvents—namely water, ethanol, ethanol:water (80:20, *v/v*), and methanol:water (80:20, *v/v*)—and the results are presented in Table 2. Extraction yields with different solvents show statistically significant differences (*p* < 0.05) between samples, which might, in part, be explained by the simultaneous extraction of soluble sugars (e.g., Spanish apple pomace, which contained the highest soluble sugar amount, reached the maximum yield when extracted with water, pure or in mixture). Likewise, each apple pomace sample showed a different extraction yield according to the selected solvent, with statistically significant differences between them. This result might, once again, be related to the soluble sugar content, since Spanish apple pomace reached the maximum yield in all cases where water was used as the extraction solvent.

Besides extraction yield, the phenolic composition of each of the prepared extracts was investigated. The tentative identification of the phenolic compounds found in the studied apple pomace extracts as well as the retention time (Rt), maximum absorbance (λ_max_), pseudomolecular ion ([M−H]^−^), and the main ion fragments (MS^2^) of each phenolic compound and their individual concentrations are presented in Table 3 and Table 4. Regarding the apple pomace samples provided by the Spanish company (Table 3), fifteen different phenolic compounds were identified: three phenolic acids (peaks **1** to **3**), four flavan-3-ols (peaks **4** to **7**), five flavonoids (peaks **9**–**11**, **13**, and **14**), and three di-hydrochalcones (peaks **8**, **12**, and **15**). Peak **1** ([M−H]^−^ at *m/z* 341) was tentatively identified as caffeic acid hexoside based on its pseudomolecular ion, while peak **2** was easily distinguished by its base peak at *m/z* 173 [quinic acid-H-H_2_O]^−^, which, according to the fragmentation pattern described by [27], allowed its tentative identification as 4-*O*-caffeoylquinic acid. Then, peaks **3**, **5**, **9**, **10**, and **13** were tentatively identified as 5-*O*-caffeoylquinic acid, epicatechin, quercetin-3-*O*-rutinoside, quercetin-3-*O*-glucoside, and isorhamnetin-3-*O*-rutinoside, respectively, by comparison with authentic standards, as well as by their MS fragmentation patterns, retention times, and UV–Vis characteristics. Peaks **4**, **6**, and **7** represent proanthocyanidin oligomers of the procyanidin class (i.e., consisting of catechin and/or epicatechin units). Peak **4** presented a pseudomolecular ion [M−H]^−^ at *m/z* 577 and MS^2^ fragmentation patterns coherent with B-type (epi)catechin dimers (i.e., (epi)catechin units with C4–C8 or C4–C6 interflavan linkages). Characteristic product ions were observed at *m/z* 451 (−126 mu), 425 (−152 mu), and 407 (−152-18 mu), attributable to the heterocyclic ring fissions (HRF), retro-Diels–Alder (RDA), and further loss of water from an (epi)catechin unit, and at *m/z* 289, which is associated with the fragment corresponding to the (epi)catechin unit. Similarly, peak **6** ([M−H]^−^ at *m/z* 865) and peak **7** ([M−H]^−^ at *m/z* 1153) can be assigned as B-type (epi)catechin trimers and tetramers, respectively. In all cases, the fragmentation patterns are coherent with those expected for these types of compounds, such as those observed for procyanidin dimers but with additional fragments from the alternative cleavages of different interflavan bonds. Peaks **11** ([M−H]^−^ at *m/z* 463) and **13** ([M−H]^−^ at *m/z* 433) were identified as quercetin derivatives, both presenting MS^2^ fragments corresponding to distinct losses of hexosyl (−162 mu) and pentosyl (−132 mu) moieties and an elution order coherent with the type of substituent sugars according to their expected polarity, although the position and nature of the sugar moieties could not be identified since their retention times did not correspond to any of the available standards. Consequently, the referred compounds were tentatively identified as quercetin-*O*-hexoside and quercetin-*O*-pentoside, respectively. Peaks **8** ([M−H]^−^ at *m/z* 583), **12** ([M−H]^−^ at *m/z* 567), and **15** ([M−H]^−^ at *m/z* 435) were tentatively identified as hydroxyphloretin-2′-*O*-xylosyl-glucoside, phloretin-2′-*O*-xyloglucoside, and phlorizin (phloretin-2′-*O*-glucoside), respectively, chalcones commonly present in apple. These compounds’ identification was performed considering previous studies describing the phenolic profile of apple pomace extracts [9].

As for the Belgian apple pomace samples that remained after cider production (Table 4), the same phenolic compound profile could only be noticed in aqueous extracts. The compounds referring to peaks **4**–**6** and **8**–**15** were not present in ethanolic extracts; **10**, **11**, **13**, and **14** were absent from MeOH:H_2_O extracts; and EtOH:H_2_O extracts did not present compounds referring to peaks **5**, **7**, **8**, **10**, **14**, and **15**. In the case of apple pomace samples from juice extraction, the results were even worse, since the chromatograms obtained from the corresponding extracts did not reveal any peaks.

In addition to their higher diversity, the extracts obtained from the Spanish apple pomace also presented the highest concentration of phenolic compounds, as was particularly obvious for EtOH (301 mg/100 g dw), MeOH:H_2_O (375 mg/100 g dw), and EtOH:H_2_O (197 mg/100 g dw) extracts. It should be remembered that apple pomace samples provided by the Spanish company were lyophilized for subsequent analysis, while the Belgian ones were subjected to thermal drying, which may be the basis of the poor results attained for the latter samples. Furthermore, the major compound found in the Spanish apple pomace extract was 5-*O*-caffeoylquinic acid, with concentrations between 66 and 114 mg/100 g dw for hydroethanolic and ethanolic extracts, respectively. However, owing to their validated cosmetic advantages linked with skin health [28], and in view of the main purpose of this work, the di-hydrochalcones (3-hydroxyphloretin-2′-*O*-xylosyl-glucoside, phloretin-2-*O*-xyloglucoside, and phloretin-2-*O*-glucoside) should also be considered. Given the presented results, it seems reasonable to indicate lyophilization as the method of choice for the pretreatment of apple pomace, as samples subjected to this drying process presented a greater number and amount of different phenolic compounds, increasing its interest for potential incorporation in dermocosmetic formulations.

### 3.3. Bioactive Properties

#### 3.3.1. Antioxidant Activity

The results obtained for antioxidant activity of the studied apple pomace extracts are presented in Table 5. Among the three studied samples extracted with different solvents, the hydromethanolic and hydroethanolic extracts from the Spanish apple pomace samples showed the highest antioxidant activity with the lowest EC_50_ values in all assays except for the β-carotene bleaching inhibition, for which the ethanolic extract proved to be more effective. These results were somehow predictable given the phenolic profile presented by the Spanish apple pomace hydromethanolic extract, which presented the greatest amount of total phenolics compared to the other studied extracts. However, when comparing different solvents, it was also evident that the antioxidant activity measured in the Spanish apple pomace extracts was provided by compounds other than phenolic compounds, as there is no direct correlation among the concentration of phenolic compounds (Table 3) and the EC_50_ values obtained in each assay.

Considering the lipid composition of human skin, the ethanolic extracts could be considered as the best choice, thus being selected for the antimicrobial activity assay described in the next section. Owing to their innocuous nature, the hydroethanolic extracts were also tested.

#### 3.3.2. Antimicrobial Activity

The antimicrobial activity of the selected ethanolic and hydroethanolic extracts from the Spanish apple pomace samples was measured against *P. acnes*, owing to their relevance in the acne process; methicillin-resistant *Staphylococcus aureus* (MRSA); *Proteus mirabilis*, and *Pseudomonas aeruginosa,* and the results are presented in Table 6. The minimal inhibitory concentration (MIC) values were not exceptionally low, but in the specific case of *P. acnes*, the results attained for the hydromethanolic extract (0.5 mg/mL) might be considered satisfactory.

### 3.4. Dermocosmetic Gel Formulation and Characterization

In order to obtain a formulation free of artificial ingredients as much as possible, pectin extracted from the apple pomace samples was employed as a gelling agent. This methodology was conducted in our lab for the first time, being especially optimized for the current work. Thus, different pH conditions were assayed, allowing to achieve pectin extraction yields varying from 1.69 up to 1.87% to be further incorporated in the formulated hydrogel.

Regarding the antimicrobial results, the hydroethanolic extract was chosen to be incorporated in the hydrogel (5 mg/mL), which was subsequently tested for its antioxidant and antimicrobial activity.

In terms of antioxidant activity, a DPPH scavenging effect of over 85% was achieved in the hydrogel formulation, while the absorbance of the reaction endpoint in the reducing power assay was 1.858, a value much higher than the established EC_50_ value (0.500).

Regarding antimicrobial activity, the formulated hydrogel was tested only against *P. acnes* as this species was the most relevant considering the purpose of this work. However, the bacterial growth was not inhibited, probably due to the 2 × dilution effect induced by the solubilization of the gel in culture medium. Therefore, a second gel was prepared with 10 mg/mL of hydroethanolic extract. In this second attempt, the growth of *P. acnes* was effectively inhibited.

## 4. Conclusions

Considering the main goal of the present study, it can be concluded that the selected drying method has a high influence over the phenolic profile, since the lyophilized samples presented a higher diversity and concentration of these compounds. Additionally, it was also verified that the chosen solvent has a considerable effect with different outcomes among thermally dried or freeze-dried samples; in fact, in the case of Spanish samples, methanol:water (375 mg/100 g dw) and ethanol (301 mg/100 g dw) led to the highest yields, while the corresponding result was obtained with water (132 mg/100 g dw) and the water:ethanol mixture (71 mg/100 g dw) in the case of Belgian samples.

Taking the former results into account, and considering the lipid composition of human skin, the hydroethanolic extract was chosen to be added to the hydrogel as it is also much less toxic than methanol.

Regarding the bioactivity of the extracts, the hydroethanolic extract presented good antibacterial activity against *P. acnes* (MIC = 2.5 mg/mL), MRSA (MIC = 2.5 mg/mL), and *Proteus mirabilis* (MIC = 10 mg/mL); the result obtained for *P. acnes* is especially important, as this bacterium is associated with skin disorders, such as acne.

For the dermal hydrogel formulation, pectin was obtained by hot acid extraction; however, this method, at an industrial scale, produces pectin with a lower degree of polymerization and produces high amounts of chemical waste. Therefore, the study of possible alternative methods, such as enzymatic extraction, would be interesting for future research in this field. The resulting hydrogel was shown to have antioxidant and antimicrobial activity against *P. acnes* at a concentration of 5 mg/mL, thus validating its application as a cosmeceutical.

In general, apple pomace proved to be a valuable source of bioactive compounds, especially polyphenols and pectin, which could be applied in dermal formulations. The apple pomace phenolics proved not only its antioxidant activity but also its antimicrobial potential against skin bacteria. However, further research would be needed in this field to confirm the anti-acne potential of apple pomace polyphenols.

Thus, it is evident that, using a circular economy approach, industrial by-products which are typically discarded as waste, could have interesting applications to obtain high-value compounds that are likely to be used in different industrial sectors, such as food, cosmetic, and pharmaceutical applications.

## Figures and Tables

**Table 1:** Proximate composition of the studied apple pomace samples (mean ± SD).

	Spanish	Belgian (Cider)	Belgian (Juice)	ANOVA (*p*-Value) (*n* = 54)
Moisture (g/100 g fw)	75.3 ± 0.4	*	*	-
Fat (g/100 g dw)	1.9 ± 0.1 ^a^	1.6 ± 0.1 ^b^	1.0 ± 0.1 ^c^	<0.001
Proteins (g/100 g dw)	3.3 ± 0.1 ^b^	2.8 ± 0.1 ^c^	4.0 ± 0.2 ^a^	<0.001
Ash (g/100 g dw)	1.7 ± 0.2 ^b^	1.4 ± 0.1 ^c^	1.9 ± 0.2 ^a^	<0.001
Carbohydrates (g/100 g dw)	93.0 ± 0.2 ^b^	94.2 ± 0.2 ^a^	93.1 ± 0.3 ^b^	<0.001
Energy value (kcal/100 g dw)	351 ± 12 ^a^	291 ± 4 ^c^	329 ± 7 ^b^	<0.001
Fructose (g/100 g dw)	46 ± 1 ^b^	42 ± 1 ^c^	55 ± 1 ^a^	<0.001
Glucose (g/100 g dw)	26 ± 2 ^a^	23 ± 1 ^b^	20 ± 1 ^c^	<0.001
Sucrose (g/100 g dw)	7.4 ± 0.2 ^a^	1.7 ± 0.1 ^b^	1.8 ± 0.1 ^b^	<0.001

Different letters across each line denote values with statistically significant differences (*p* < 0.05). The calibration curve equations were as follows: fructose (y = 1.04x; R^2^ = 0.999; LOD = 0.05 mg/mL; LOQ = 0.18 mg/mL); glucose (y = 0.935x; R^2^ = 0.999; LOD = 0.08 mg/mL; LOQ = 0.25 mg/mL); sucrose (y = 1.17675x; R^2^ = 0.997; LOD = 0.06 mg/mL; LOQ = 0.30 mg/mL). Energy values were calculated as follows: m_fat_ × 9 + m_(proteins + fructose + glucose + sucrose)_ × 4 (where m values were included in grams). * Samples were provided dried.

**Table 2:** Phenolic compounds’ extraction yields from the studied apple pomace decoction, ethanolic, hydromethanolic, and hydroethanolic extracts (relative %; mean ± SD).

	H_2_O	EtOH	EtOH:H_2_O (80:20, *v/v*)	MeOH:H_2_O (80:20, *v/v*)	ANOVA (*p*-Value) (*n* = 54)
Spanish	52 ± 2 ^aA^	44 ± 1 ^bC^	47 ± 3 ^bB^	47 ± 1 ^bB^	<0.001
Belgian (cider)	28 ± 1 ^cB^	41 ± 1 ^bA^	41 ± 3 ^cA^	40 ± 1 ^cA^	<0.001
Belgian (juice)	37 ± 2 ^bC^	65 ± 8 ^aA^	52 ± 2 ^aB^	50 ± 1 ^aB^	<0.001
ANOVA (*p*-value) (*n* = 54)	<0.001	<0.001	<0.001	<0.001	-

Different lowercase letters and uppercase letters across each line denote values with statistically significant differences (*p* < 0.05) for at least one apple pomace type or for at least one solvent, respectively.

**Table 3:** Retention time (Rt), wavelengths of maximum absorption in the visible region (λ_max_), mass spectral data, identification, and quantification of phenolic compounds in apple pomace from Corporación Hijos de la Rivera S.L. (Spain).

Peak	Rt (min)	λ_max_ (nm)	Molecular Ion (*m/z*)	MS^2^ (*m/z*)	Tentative Identification	Quantification (mg/100 g dw)				ANOVA (*p*-Value) (*n* = 72)
						EtOH	H_2_O	MEOH:H_2_O	EtOH:H_2_O	
1	4.87	324	341	179(100)	Caffeic acid hexoside	tr	tr	tr	tr	-
2	6.67	322	353	191(12), 179(1), 173(100), 161(1), 135(2)	4-*O*-Caffeoylquinic acid	14 ± 2 ^c^	20 ± 1 ^a^	18 ± 2 ^b^	11 ± 2 ^d^	<0.001
3	7.24	327	353	191(100), 179(6), 173(2), 161(1), 135(1)	5-*O*-Caffeoylquinic acid	114 ± 20 ^a^	69 ± 6 ^b^	108 ± 16 ^a^	66 ± 1 ^b^	<0.001
4	8.02	281	577	451(24), 425(100), 407(21), 289(12)	B-type (epi)catechin dimer	18 ± 4 ^b^	16 ± 1 ^b^	29 ± 6 ^a^	19 ± 4 ^b^	<0.001
5	9.82	281	289	245(100), 203(5), 187(1), 161(2), 137(2)	Epicatechin	31 ± 1	tr	12 ± 1	tr	<0.001 *
6	11.39	280	865	739(74), 713(44), 695(100), 577(64), 575(37), 425(10), 407(9), 289(8), 287(7)	B-type (epi)catechin trimer	10 ± 2 ^b^	10 ± 1 ^b^	17 ± 1 ^a^	7 ± 2 ^c^	<0.001
7	12.54	280	1153	865(19), 863(18), 577(6), 575(11), 289(3), 287(4)	B-type (epi)catechin tetramer	2.4 ± 0.2 ^d^	4.0 ± 0.1 ^c^	15 ± 2 ^a^	7 ± 2 ^b^	<0.001
8	16.13	282	583	289(100)	3-Hydroxyphloretin-2′-*O*-xylosyl-glucoside	4.5 ± 0.5 ^b^	1.9 ± 0.4 ^d^	3.6 ± 0.4 ^c^	5.6 ± 0.5 ^a^	<0.001
9	17.95	353	609	301(100)	Quercetin-3-*O*-rutinoside	13 ± 1 ^c^	21 ± 1 ^b^	24 ± 2 ^a^	12 ± 1 ^c^	<0.001
10	18.79	354	463	301(100)	Quercetin-3-*O*-glucoside	13 ± 1 ^b^	nd	24 ± 3 ^a^	13 ± 1 ^b^	<0.001
11	19.14	353	463	301(100)	Quercetin-*O*-hexoside II	13 ± 1 ^b^	nd	24 ± 1 ^a^	12 ± 1 ^b^	<0.001
12	19.70	285	567	273(100)	Phloretin-2-*O*-xyloglucoside	20 ± 4 ^a^	tr	15 ± 1 ^b^	11 ± 1 ^c^	<0.001
13	21.84	356	433	301(100)	Quercetin-*O*-pentoside	12 ± 1 ^c^	20 ± 1 ^b^	22 ± 1 ^a^	11 ± 1 ^d^	<0.001
14	22.27	350	623	315(100)	Isorhamnetin-3-*O*-rutinoside	12 ± 1 ^c^	20 ± 1 ^b^	24 ± 2 ^a^	12 ± 1 ^c^	<0.001
15	22.89	285	435	273(100)	Phlorizin (phloretin-2-*O*-glucoside)	24 ± 1 ^b^	tr	39 ± 1 ^a^	9 ± 1 ^c^	<0.001
					Phenolic compounds	301 ± 32 ^b^	182 ± 7 ^c^	375 ± 42 ^a^	197 ± 5 ^c^	<0.001

tr: traces; nd: not detected. In each line, different letters represent statistically significant differences (*p* < 0.05) for at least one solvent. * In this case, differences were evaluated by Student’s *t*-test as only two groups of results were available. Calibration curves: 1–caffeic acid (y = x + 406369; *R^2^* = 0.994; LOD = 0.78 μg/mL; LOQ = 1.97 μg/mL); 2–chlorogenic acid (y = 168823x − 161172; R^2^ = 0.9999; LOD = 0.20 µg/mL; LOQ = 0.68 µg/mL); 3–(+)-catechin (y = 84950x − 23200; R²= 1; LOD = 0.17 μg/mL; LOQ = 0.68 μg/mL); 4–isoliquiritigenin (y = 42820x + 184902; *R^2^* = 0.9999; LOD = 0.18 µg/mL; LOQ = 0.54 µg/mL); 5–quercetin 3-*O*-glucoside (y = 34843x − 160173; *R^2^* = 0.9998; LOD = 0.21 µg/mL; LOQ = 0.71 µg/mL).

**Table 4:** Retention time (Rt), wavelengths of maximum absorption in the visible region (λ_max_), mass spectral data, tentative identification, and quantification of phenolic compounds in apple pomace from Tuinsappen Lombarts Calville (Belgium).

Peak	Rt (min)	λ_max_ (nm)	Molecular Ion (m/z)	MS^2^ (m/z)	Tentative Identification	Quantification (mg/100 g dw)				ANOVA (*p*-Value) (*n* = 72)
						EtOH	H_2_O	MEOH:H_2_O	EtOH:H_2_O	
1	4.87	364	341	179(100)	Caffeic acid hexoside	0.64 ± 0.02	tr	tr	tr	-
2	6.67	322	353	191(12), 179(1), 173(100), 161(1), 135(2)	4-*O*-Caffeoylquinic acid	4.1 ± 0.3 ^c^	15 ± 4 ^a^	10 ± 1 ^b^	9 ± 2 ^b^	<0.001
3	7.24	327	353	191(100), 179(6), 173(2), 161(1), 135(1)	5-*O*-Caffeoylquinic acid	2.7 ± 0.3 ^c^	12 ± 3 ^a^	9 ± 1 ^b^	7 ± 1 ^b^	<0.001
4	8.02	281	577	451(24), 425(100), 407(21), 289(12)	B-type (epi)catechin dimer	nd	nd	3.3 ± 0.4	5.1 ± 0.5	<0.001 *
5	9.82	281	289	245(100), 203(5), 187(1), 161(2), 137(2)	Epicatechin	nd	tr	tr	nd	-
6	11.39	280	865	739(74), 713(44), 695(100), 577(64), 575(37), 425(10), 407(9), 289(8), 287(7)	B-type (epi)catechin trimer	nd	8 ± 2 ^a^	3.2 ± 0.3 ^c^	6 ± 2 ^b^	<0.001
7	12.54	280	1153	865(19), 863(18), 577(6), 575(11), 289(3), 287(4)	B-type (epi)catechin tetramer	2.6 ± 0.2 ^b^	4.4 ± 0.5 ^a^	3.2 ± 0.2 ^b^	nd	<0.001
8	16.13		583	289(100)	3-Hydroxyphloretin-2′-*O*-xylosyl-glucoside	nd	tr	tr	nd	-
9	17.95	353	609	301(100)	Quercetin-3-*O*-rahamnoside	nd	18 ± 1 ^a^	18 ± 1 ^a^	15 ± 2 ^b^	<0.001
10	18.79	354	463	301(100)	Quercetin-*O*-hexoside I	nd	18 ± 1	nd	nd	-
11	19.14	353	463	301(100)	Quercetin-*O*-hexoside II	nd	19 ± 1	nd	15 ± 3	<0.001 *
12	19.70	285	567	273(100)	Phloretin-2-*O*-xyloglucoside	nd	tr	tr	tr	-
13	21.84	356	433	301(100)	Quercetin-*O*-pentoside	nd	19 ± 1	nd	15 ± 2	<0.001 *
14	22.27	350	623	315(100)	Isorhamnetin-3-*O*-rutinoside	nd	18 ± 1	nd	nd	-
15	22.89	285	435	273(100)	Phlorizin (phloretin-2-*O*-glucoside)	nd	tr	tr	nd	-
					Phenolic compounds	10.0 ± 0.4 ^d^	132 ± 12 ^a^	47 ± 3 ^c^	71 ± 6 ^b^	<0.001

tr: traces; nd: not detected. In each line, different letters represent statistically significant differences (*p* < 0.05) for at least one solvent. *In this case, differences were evaluated by the t-Student test, as only two groups of results were available. Calibration curves: 1–caffeic acid (y = 388345x + 406369; *R^2^* = 0.994; LOD = 0.78 μg/mL; LOQ = 1.97 μg/mL); 2- chlorogenic acid (y = 168823x − 161172; R^2^ = 0.9999; LOD = 0.20 µg/mL; LOQ = 0.68 µg/mL); 3–(+)-catechin (y = 84950x − 23200, R²= 1; LOD = 0.17 μg/mL; LOQ = 0.68 μg/mL); 4- isoliquiritigenin (y = 42820x + 184902; *R^2^* = 0.9999; LOD = 0.18 µg/mL; LOQ = 0.54 µg/mL); 5–quercetin 3-*O*-glucoside (y = 34843x − 160173; *R^2^* = 0.9998; LOD = 0.21 µg/mL; LOQ = 0.71 µg/mL).

**Table 5:** Antioxidant activity of the studied apple pomace samples (EC_50_, µg/mL; mean ± SD).

**DPPH Scavenging Activity**				
	**EtOH**	**H_2_O**	**MeOH:H_2_O (80:20)**	**EtOH:H_2_O (80:20)**
Spanish	4.1 ± 0.4	1.7 ± 0.2	0.6 ± 0.1	0.7 ± 0.1 ^c^
Belgian (cider)	>10	9 ± 1	>10	10 ± 1 ^a^
Belgian (juice)	>10	>10	>10	9 ± 1 ^b^
ANOVA or Student’s *t*-test (*p*-value) (*n* = 54)	-	<0.001	-	<0.001
**Reducing Power**				
	**EtOH**	**H_2_O**	**MeOH:H_2_O (80:20)**	**EtOH:H_2_O (80:20)**
Spanish	1.0 ± 0.1	1.0 ± 0.1	0.35 ± 0.02	0.62 ± 0.02 ^c^
Belgian (cider)	>10	4.2 ± 0.5	>10	5.2 ± 0.3 ^a^
Belgian (juice)	>10	>10	>10	2.5 ± 0.1 ^b^
ANOVA or Student’s *t*-test (*p*-value) (*n* = 54)	-	<0.001	-	<0.001
**β-Carotene Bleaching Inhibition**				
	**EtOH**	**H_2_O**	**MeOH:H_2_O (80:20)**	**EtOH:H_2_O (80:20)**
Spanish	0.8 ± 0.1	3.4 ± 0.3	1.4 ± 0.2	1.2 ± 0.3
Belgian (cider)	>10	>10	>10	>10
Belgian (juice)	>10	>10	>10	>10
ANOVA (*p*-value) (*n* = 54)	-	-	-	-

Results of the antioxidant activity are expressed in EC_50_ values: sample concentration providing 50% of the antioxidant activity or 0.5 of absorbance in the reducing power. Uppercase letters indicate statistically significant differences among each pomace type (different classifications for each antioxidant assay).

**Table 6:** Antimicrobial activity of the selected apple pomace ethanolic and hydroethanolic extracts from Corporación Hijos de la Rivera S.L. (Spain).

	EtOH Extract	EtOH:H_2_O Extract
Bacteria	MIC (mg/mL)	MIC (mg/mL)
*Propionibacterium acnes*	5	2.5
MRSA	5	2.5
*Proteus mirabilis*	20	10
*Pseudomonas aeruginosa*	>20	>20

Minimum inhibitory concentration (MIC); methicillin-resistant *Staphylococcus aureus* (MRSA).
